# Heat stress in poultry: the role of nutritional supplements in alleviating heat stress and enhancing gut health in poultry

**DOI:** 10.3389/fvets.2025.1691532

**Published:** 2025-11-06

**Authors:** Sammad F. Olayiwola, Sunday A. Adedokun

**Affiliations:** Department of Animal and Food Sciences, University of Kentucky, Lexington, KY, United States

**Keywords:** gut health, heat stress, nutritional supplements, oxidative stress, performance, poultry

## Abstract

Globally, heat stress (HS) is a major concern in poultry farming, adversely impacting bird productivity, health, welfare, and economic returns. As climate change intensifies, the occurrence and severity of HS are anticipated to rise, posing greater risks to the poultry industry and the increasing demand for food. Birds respond to HS by exhibiting different mechanisms, including behavioral and physiological changes, to regulate their body temperature. In poultry, HS has been associated with reduced feed consumption, growth, feed efficiency, quantity and quality of eggs produced, meat quality, reproductive performance, impaired gut health, and increased mortality. Also, HS induces acid–base imbalance, causing both respiratory alkalosis and metabolic acidosis. During HS, birds pant to cool down and exhale excessive carbon dioxide, leading to a decrease in blood pH. Nutritional interventions have emerged as a viable strategy to mitigate HS effects, with various dietary supplements demonstrating efficacy in improving poultry resilience. Vitamins (A, C, D, and E), minerals (selenium, zinc, chromium, sodium, potassium, and chloride), fat, amino acids, electrolytes, and *in ovo* feeding have been revealed to boost thermotolerance, support growth, and improve feed efficiency of birds under HS conditions. This review integrates current literature on the impact of HS on poultry production and examines how nutritional supplements can help alleviate the effects of this environmental stressor in the avian species.

## 1 Introduction

### 1.1 Significance of global poultry production

**Figure 1:**
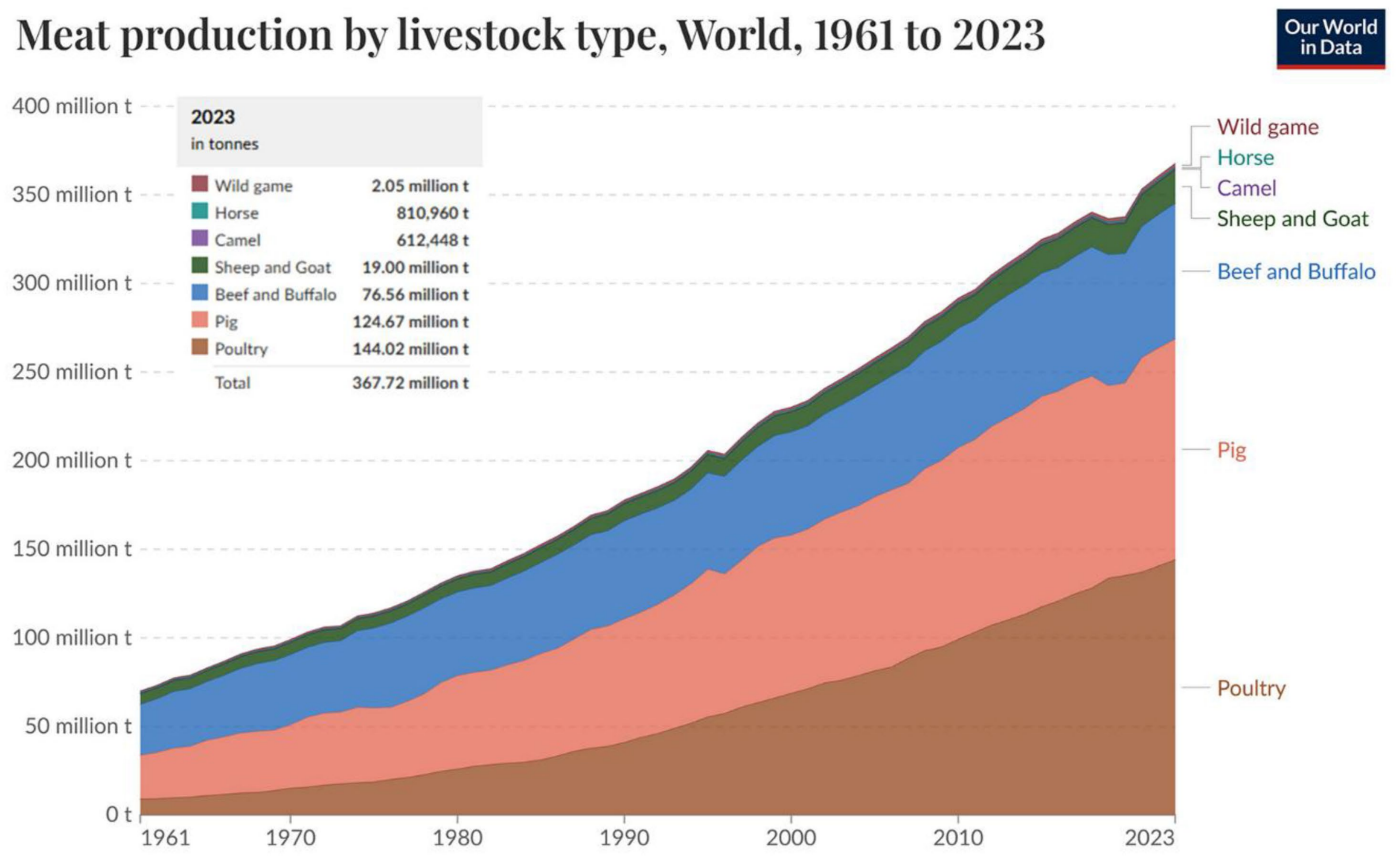
Livestock meat production from commercial and farm slaughter based on dressed carcass weights and slaughter fats. Source: Adapted from FAO (4).

The contribution of poultry to the global economic and food system cannot be overstated. Poultry meat and eggs are widely consumed globally, providing a readily available and affordable source of high-quality protein, essential vitamins (A, B_2_, and B_12_), and important minerals (calcium, zinc, and iron), thereby proving to be essential for healthy human nutrition (1). Also, poultry products (meat and eggs) have no cultural or religious taboos (2), which contribute to their widespread global acceptance and consumption (3). Globally, 144.02 million tons of meat is generated from poultry (Figure 1), accounting for over one-third of total meat produced (4). In 2024, the United States generated $70.2 billion from the sales of poultry products, including eggs from laying hens and meat from broilers and turkey breeders (5). Also, the generally low cost of poultry products makes them an accessible source of nutrition for individuals in both developing and developed countries, ultimately supporting food security and improving livelihoods (6). This highlights the vital role of poultry products in the global economy, supporting food security, rural development, and poverty alleviation, especially in developing nations (7).

In recent years, the demand for more animal-derived protein has risen due to population growth, increasing income, and urbanization (8), leading to a rapid increase in global poultry production (7). The increase in the poultry industry’s growth is favored by its products’ affordability and short generational interval (9). Likewise, research advancements in genetics, nutrition, disease control, and housing management have contributed to this growth (6). For example, compared to other livestock, broilers have evolved to have a relatively short generational interval and high feed efficiency, making them a highly efficient source of affordable protein for the expanding global population (10). Also, laying hens have been genetically selected and improved over time for longer production cycles, thereby increasing their contribution to the supply of animal protein. Economically, the poultry industry supports millions of livelihoods (11), particularly in developing countries, where small-scale and backyard farming provide both food and income. From an ecological perspective, poultry farming generates a lower environmental footprint than other livestock, like cattle and swine (12).

### 1.2 Stress

Poultry is one of the fastest-growing sources of animal protein (7). Nevertheless, poultry production faces numerous challenges, including stress, high feed cost, and disease outbreaks, which threaten the performance, efficiency, sustainability, and profitability of poultry production. Selye (13) articulated the concept of stress as “the nonspecific response of the body to any demand,” while a stressor was considered as “an agent that induces stress at any given time.” Birds are vulnerable to various stressors, including environmental stressors (heat and cold stress, light, air quality, and humidity), management stressors (litter quality, stocking density, poor ventilation, beak trimming, vaccination, transportation, and handling), nutritional stressors (nutrient deficiencies, feed contamination, and mycotoxins), and biological or internal stressors such as diseases and gut microbiota imbalances (14). Poultry exposed to stress experience a disruption in their normal physiological homeostasis.

Physiologically, stress occurs when there is a shift away from ideal internal and external conditions. During stress, the hypothalamic–pituitary–adrenal (HPA) axis, the immune system, and the autonomic nervous system work together to restore homeostatic balance. Exposure to stress induces a series of regulatory mechanisms in the body, causing metabolic adjustments such as heightened energy mobilization and metabolic shifts that impair poultry performance (15). Modern poultry management practices, including optimized nutrition and environmental control, have greatly reduced the incidence of stress-induced nutritional diseases like encephalomalacia and muscular dystrophy (16). Nevertheless, stress continues to adversely affect poultry productivity and reproduction, leading to considerable economic losses.

This review identified heat stress (HS) as an environmental stressor, focusing on its impacts on poultry performance, productivity, and health. It also discusses the efficacy of nutritional supplements like vitamins, minerals, and amino acids to alleviate HS and support gut health in poultry. By linking these dietary strategies to improved gut function, resilience, and productivity, the review provides practical insights for optimizing poultry health and performance while identifying areas for future research.

## 2 Thermal stress in poultry

**Figure 2:**
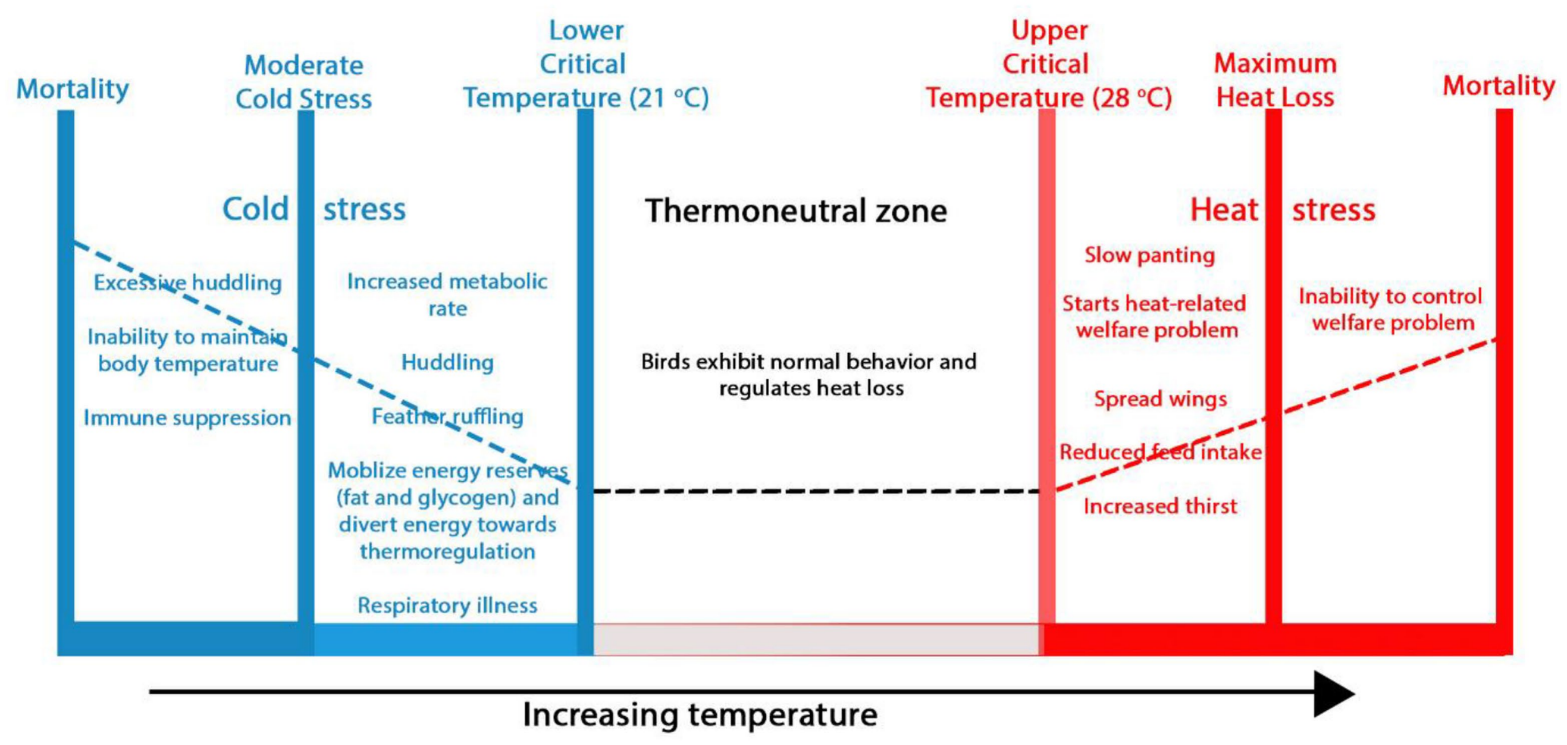
Impact of ambient temperature on poultry behavior and welfare. Within the thermoneutral zone, birds maintain a stable body temperature through physical heat regulation and display normal behaviors. Beyond the lower and upper critical zones, chickens experience cold and heat stress, respectively, leading to welfare issues and even death.

Similar to other warm-blooded animals, poultry are homeothermic and maintain a relatively constant body temperature through a complex thermoregulatory system that balances heat production and dissipation. This balance is most efficient within a thermoneutral zone of 21°C to 28°C (17), where birds are comfortable and can balance the amount of heat generated and lost by the body (Figure 2). However, the internal temperature of poultry species can vary with size, breed, and sex, making them exhibit different responses to temperature outside the thermoneutral zone. When environmental conditions (temperature and humidity) exceed the birds’ thermal comfort zone, their capacity to regulate body temperature is compromised, resulting in HS. Different variants of birds show varying levels of resistance to HS, with the fast-growing breeds exhibiting significantly reduced resistance (18). Moreover, the susceptibility of poultry, especially broilers, to HS is compounded by their inherent physiological characteristics, including a high metabolic rate that generates significant internal heat and the absence of sweat glands to effectively disperse heat through sweating, which is a primary mechanism utilized by other species like horses, donkeys, and camels (17, 19).

### 2.1 Mechanism of heat stress

**Figure 3:**
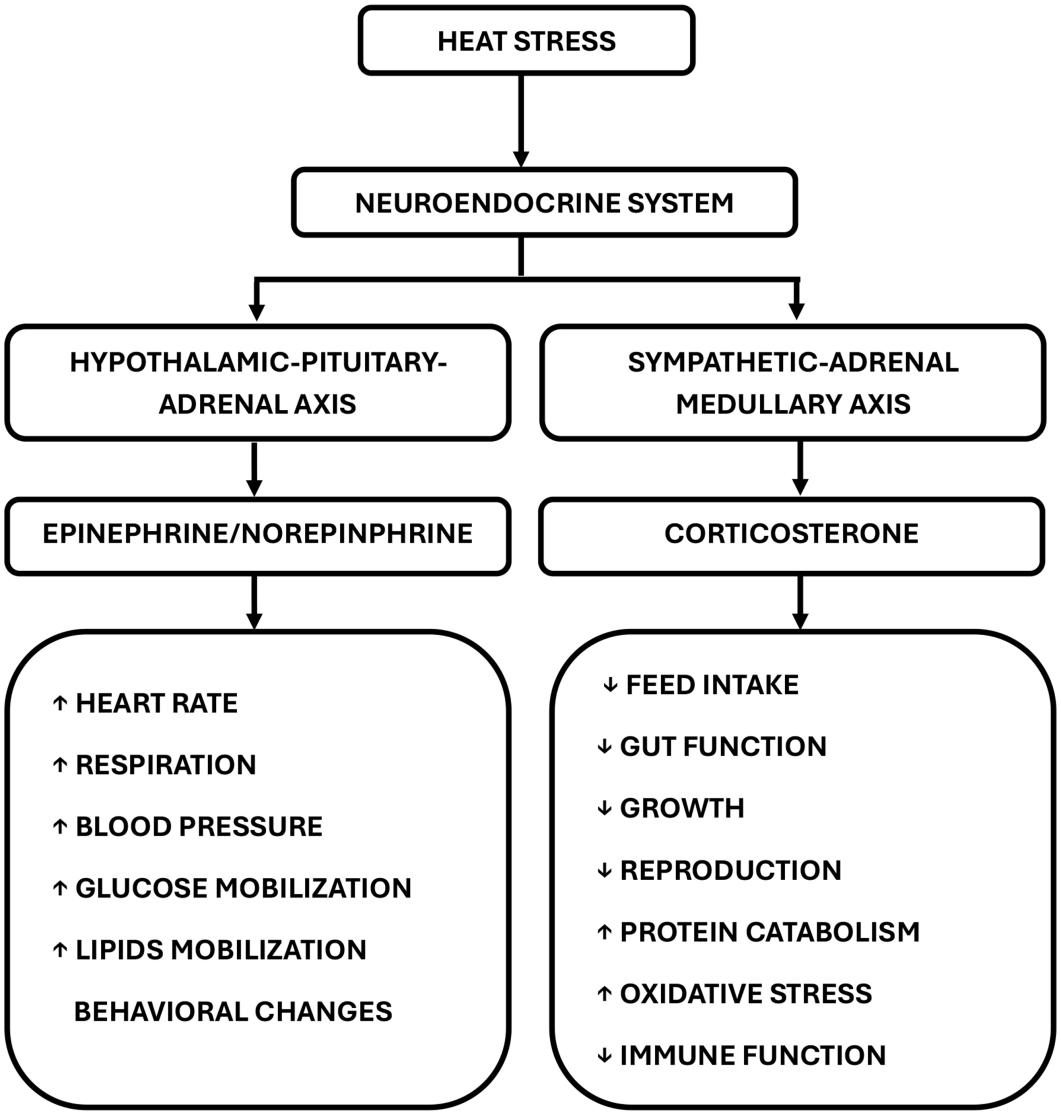
Mechanism of heat stress. Heat stress stimulates the hypothalamic–pituitary–adrenal and sympathetic-adrenal medullary axis to generate several physiological effects. The secretion of epinephrine or norepinephrine increases respiration, heart rate, blood pressure, glycogenolysis, and lipolysis. The release of corticosterone compromises the immune system, reduces feed intake, and impairs gut function.

Poultry respond differently to HS, depending on the heat intensity and duration of exposure. The neuroendocrine system is pivotal in sustaining optimal physiological functions in living organisms. Elevated ambient temperatures impact the neuroendocrine system by activating the sympathetic-adrenal medullary (SAM) and the HPA axes (Figure 3) (20), which are the primary pathways for modifying the immune response (21). This activation promotes increased glucose synthesis, which is vital for the survival of chickens under stressful conditions (22). The SAM regulates the fight against cell invasion by detecting stimuli and transferring information from the hypothalamus to the adrenal gland (23). In stressful situations, the SAM secretes catecholamines, like norepinephrine and epinephrine (adrenaline), triggering a rapid response characterized by an increased heart rate and glucose production (24).

The presence of primary glucocorticoids (cortisol and corticosterone) varies among species, with corticosterone predominantly found in avians and rodents, while cortisol is more commonly observed in ruminants, swine, and fish (25). Corticosterone is released from the HPA axis and the pituitary gland through the action of adrenocorticotropic hormone (ACTH) (26). Unlike adrenaline, corticosterone is secreted more slowly, leading to sustained physiological effects (25), making it a dependable stress indicator (21). In chickens, persistent corticosterone secretion is associated with cardiovascular diseases, a compromised immune system, depression, and muscle breakdown due to gluconeogenesis and reduced cognition (26).

### 2.2 Poultry responses to heat stress

#### 2.2.1 Regulation of body temperature

Under HS, birds regulate their body temperature through the hypothalamus because they lack sweat glands. At first, birds disperse heat to the environment through conduction (transfer of body heat to cooler surfaces in direct contact), convection (loss of heat to surrounding air as warmer air near the body surface rises and is replaced by cooler air), and radiation, which is the emission of heat in the form of infrared energy from the body surface to cooler objects in the environment (17, 27). This thermoregulation system functions optimally within the thermoneutral range, and the size of the comb and wattle significantly contributes to this mechanism. These structures provide the body with ample bare skin to facilitate blood circulation, leading to the dissipation of heat from the body to the head and losing this heat more efficiently. Approximately 40% of the heat that the bird seeks to dissipate is lost through these mechanisms (17). As environmental temperatures hit the upper critical threshold (>26–29°C), birds change their heat loss mechanism to evaporative processes (28). The evaporative mechanism involves thermal exhaustion and intensified evaporative cooling, resulting from increased blood flow to the skin, elevated heart rate, and dilation of peripheral blood vessels. This mechanism is facilitated by the air sacs, lungs, and mucous area between the nasal openings and the tracheal base through increased gaseous exchange and air circulation on surfaces (17, 21, 28).

#### 2.2.2 Behavioral responses

As temperature rises further, birds exhibits various behavioral strategies to regulate their body temperature and mitigate the effects of HS. Under HS, birds often become exhausted, lose their appetite, spread their wings away from their bodies, pant to increase their respiratory rate and facilitate heat dissipation, and seek cooler environments (17). Heat-stressed birds modify their feeding and activity patterns with dramatic reductions in feed intake (FI) during hot periods (29). This response is a strategy aimed at lowering metabolic heat production, as feed digestion, absorption, and nutrient utilization generate additional heat; therefore, eating less helps limit their internal heat production (30). While beneficial for thermoregulation, reduced FI directly impacts growth and egg production. To compensate for water lost through panting and to maintain hydration, chickens often increase their water intake.

Additionally, heat-stressed birds become notably lethargic, spending more time resting on the ground (to maximize heat transfer to the cooler floor) and less time moving. Activities are minimized to reduce metabolic heat production from physical activities, and blood flow is redirected from internal organs to the skin to facilitate heat dissipation (31). In flocks, birds may spread out to avoid crowding (seeking space for better airflow) or seek cooler microclimates within their environment (17, 31). Behavioral signs of discomfort can include panting, drooping wings, ruffled feathers, and sometimes mild aggression (pecking in laying hens) or restlessness as they seek relief (32). Some of these behaviors consume energy; for instance, panting and wing-flapping require muscular effort, which slightly increases maintenance energy expenditure (33). Consequently, prolonged panting diverts energy from growth or egg production toward combating HS, leading to performance loss.

#### 2.2.3 Physiological responses

The physiological response to HS in birds is mediated by the hypothalamus, pituitary gland, and adrenal gland. High humidity limits evaporative and convective cooling, thereby increasing the heat load on the bird (34). Notably, modern fast-growing broilers are prone to HS (35) due to their high metabolic heat production and relatively underdeveloped cardio-respiratory capacity (36). Fast-growing broilers with higher body weights show more severe hyperthermia and lower heat tolerance than smaller and slow-growing breeds under the same conditions (37). In both broilers and layers, prolonged heat exposure raises the internal body temperature beyond the thermoneutral range, resulting in systemic disturbances such as dehydration (due to panting-related water loss) and electrolyte imbalances.

High temperatures affect poultry’s neuroendocrine system, triggering the HPA axis to release corticotropin-releasing factor (CRF) from the hypothalamus (38). This signals the pituitary gland to release ACTH and stimulates the adrenal cortex to produce corticosteroids, which increase electrolyte and bicarbonate losses (17). Elevated corticosterone is a hallmark of acute HS response in chickens and chronically heat-stressed birds often show persistently high corticosterone levels. This hormonal shift has multiple downstream effects, including induced muscle proteolysis, suppressed protein synthesis, and increased lipogenesis (fat deposition) (33). These catabolic effects partly explain the reduction in lean muscle growth and increased abdominal fat often observed in heat-stressed broilers (33). Elevated plasma concentrations of corticosteroids have been shown to be immunosuppressive (39), modifying glucose production and mineral metabolism, which contributes to the pathogenesis of gastrointestinal lesions, cardiovascular disorders, hypercholesterolemia, and alterations in immune function in heat-stressed birds (40).

In addition to adrenal hormones, thyroid hormones are markedly affected by HS. The homeostasis of triiodothyronine (T_3_) and thyroxine (T_4_), is important for regulating body temperature and metabolism (28). Although the impact of high temperature on T_4_ levels varies, the concentration of T_3_ levels in the blood reduces under high temperature (41, 42). Literature has reported a reduction in both T_3_ and T_4_ concentrations in heat-stressed broilers (43, 44) and laying hens (45). The hypothyroid response is thought to be an adaptive mechanism to lower basal metabolic rate and decrease metabolic heat production during chronic HS (46). Reducing T_3_ and T_4_ levels may decrease internal heat production, but it leads to slower growth (47), increased carcass fat from lower lipid metabolism (48), and poorer egg production and eggshell quality in laying hens (49). Decreased thyroid activity can partially explain the growth depression and poorer eggshell calcification observed during HS. Additionally, the thyroid gland is crucial in initiating puberty and regulating reproductive functions in avian species. Therefore, a disruption to thyroid activity due to HS would adversely affect hens’ reproductive performance (42).

## 3 Effects of heat stress on productivity traits in poultry

### 3.1 Impact of heat stress on growth and meat quality

The effects of HS on bird strains and gender, as well as FI and body weight gain (BWG), are shown in Tables 1, 2, respectively. Broilers generate a large amount of heat because of the high energy density in their diets, retaining only 40% of the ingested energy and dissipating the remaining 60% (17). Exposing broilers to chronic HS resulted in 16.4, 32.6, and 25.6% reductions in FI, BWG, and feed conversion ratio (FCR), respectively (28, 50). According to Harsini et al. (51), exposing broilers to a cyclic temperature range of 23.9°C to 37°C significantly reduced BWG and FI, and increased FCR. Likewise, HS in laying hens led to reduced body weight (BW), feed efficiency, and other production metrics (52). The degree to which FI decreases varies based on different factors linked to the HS model applied to birds, complicating comparisons across studies (53). Under HS, birds reduce their FI to allocate more energy to cooling efforts, diverting energy from growth to thermoregulation. In addition to reduced FI, HS reduces the blood supply to intestinal epithelial cells, thereby reducing nutrient and oxygen supply, which in turn causes morphological changes and mucosal damage. This leads to intestinal inflammation, reduced villus height, and intestinal permeability, which impairs nutrient absorption and further contributes to poor feed efficiency and growth performance (54).

**Table 1:** Effects of heat stress on bird strains and gender.

Strain	Age	Sex	Housing condition	Impacts	References
Hubbard-Cobb	42 days	Male and female	TN: 21–22°C and 60% RH HS: 35°C and 60% RH for 8 h/day	The heat-stressed birds had higher mortality (11.5%) than the TN birds (5.2%) Heat stress significantly increased ionized sodium concentration and decreased ionized potassium concentration Total protein, uric acid, and globulin levels were reduced in the heat-stressed birds compared to those in TN	(267)
Cobb 500	42 days	Male	TN: 24°C HS: 34°C	Heat stress reduced FBW, BWG, and FI by 20, 29, and 16%, respectively Heat stress increases FCR Compared to the TN group, HS increased the heterophils to lymphocytes ratio (0.79 vs. 0.39), corticosterone (6.49 vs. 2.00 ng/mL), TNF-*α* (166.95 vs. 98.39 pg./mL), and malondialdehyde (3.89 vs. 1.10 μmol/mL) Compared to TN, HS increased TP, AST, and ALT by 46, 65, and 95%, respectively, while triiodothyronine decreased by 43% Heat stress inhibited the immune response of broilers when compared to broilers in the TN group by decreasing the total white blood cell (34.03 vs. 55.34 × 10^3^/mL), T-lymphocyte (3.32 vs. 6.29 stimulation index (SI)), B-lymphocyte (1.62 vs. 2.84 SI), and anti-SRBC AB titer (3.93 vs. 6.95 log_2_)	(268)
Cobb 500	42 days	Male	HS: 35 °C for 8 h/day TN: 24°C	Heat stress altered growth performance by reducing FI and BW Heat stress raised the broiler’s core body temperature by 0.5°C Heat stress significantly lowered body part weights, including the hot carcass without giblet, chilled carcass without giblet, breast, tender, wings, and leg Heat stress significantly reduced the breast meat yellowness at Processing	(269)
Ross 308	42 days	Male	TN: 25°C Heat-challenged: 30°C	Compared to the control, HS reduced the FBW and FI by 19.0 and 10.4%, respectively Mortality was higher in the heat-stressed birds (10.0%) than in the TN birds (4.9%)	(270)
Laying hens (strain not mentioned)	58 weeks	Female	32°C; 50% RH 27°C; 50% RH 32°C; 50% RH	Heat stress reduced the bird’s laying performance and egg quality Heat stress impacts the physiological parameters by altering the gut metabolites and mineral/lipid metabolism	(67)
Cobb	35 days	Undefined	TN: 20°C HS: 27.8 °C	Heat stress decreased growth performance by lowering FBW (9.3%), BWG (17.9%), and FI (5.0%) HS increased intestinal permeability by decreasing the jejunal trans-epithelial electric resistance (TER)	(271)
Ross 308	42 days	Undefined	35 ± 2°C from 15 to 42 days of age	Heat stress lowers FI (15.2%), BWG (19.2%), and deteriorates FCR (1.91 vs. 1.81) when compared with those kept in the thermoneutral house Heat stress reduced antibody responses against Newcastle disease and infectious bronchitis virus Heat stress reduces the proportion of helper (CD4+) T lymphocytes and increases cytotoxic (CD8+) T lymphocytes, leading to a lower CD4 + to CD8 + ratio in peripheral blood circulation	(225)
Ross 308	42 days	Male and female	TN: 24°C HS: 34°C	Heat stress caused a decrease in FBW (7.0%) and feed consumption (5.1%), while FCR increased by 2.0%. The reduced FBW was greater in males (8.3%) than in females (2.8%) Heat stress reduced BWG in males (8.4%) and females (2.9%) Heat stress raised the pH of broiler drumsticks and breast meat Heat stress adversely impacted the microbiological quality of meat	(272)
El-Salam (a white feathers crossbred)	84 days	Male and female	chronic HS: 38°C; 60% RH for 4 h for 3 successive days weekly	growth performance as impaired by chronic heat stress Heat stress decreased BWG and FI by 11.1 and 4.8%, respectively Heat stress increased FCR Chronic heat stress reduced the spleen percentage relative to TN Chronic heat stress reduced dressing percentage, liver, giblets, and meat moisture compared to TN Chronic heat stress increased the blood pH, plasma triglycerides, and total serum calcium, while decreasing PCV, HGB, total serum protein, and plasma glucose compared to TN Heat stress increases rectal temperature and respiration rate	(273)
Broiler (strain not mentioned)	50 days	Male and female	TN: 21 ± 1°C HS: 31 ± 1°C	Heat stress decreased the digestibility of most amino acids in females but not in males	(69)

ALT, alanine aminotransferase; AST, aspartate aminotransferase; BW, body weight; BWG, body weight gain; FBW, final body weight; FCR, feed conversion ratio; FI, feed intake; HGB, hemoglobin; HS, heat stress; PCV, packed cell volume; RH, relative humidity; TN, thermoneutral; TP, total protein.

**Table 2:** Effects of heat stress on feed intake and body weight gain of poultry.

Specie	Age	Heat stress condition	Δ in feed intake	Δ in body weight gain	Alkalosis or acidosis	References
Broiler	42 days	Chronic high ambient temperature (35 ± 2°C)	↓ 16.4%	↓ 32.6%		(50)
Laying hen	32 weeks	Acute HS: 33°C; 66% RH	↓ 30%	N/A (BW change not primary metric for layers) egg production ↓ 11%	Metabolic acidosis (↑K^+^, ↓Na^+^ are more consistent with compensated or decompensated metabolic acidosis)	(86)
Turkey	35 days	Control: 32°C and 60% RH Cold stress: 29°C and 60% RH Heat stress: 35°C and 60% RH Heat stress plus humidity: 35°C and 75% RH	No significant change	No significant change	Respiratory alkalosis (elevated blood pH and low pCO_2_ observed under HS, indicating panting)	(274)
Turkey	24 weeks	Chronic HS: 35°C	Not reported	↓ 13%		(275)
Quail	38 weeks	Cyclic HS: 36°C for 12 h/day	↓ 21.6%	N/A (BW change not primary metric for layers) egg production ↓ 6.7%		(82)
Broiler	35 days	TN: 24°C HS: 34°C	↓ 5.1%	↓ 8.4% in males ↓ 2.9% in females		(272)
Broiler	42 days	TN: 24°C HS: 34°C	↓ 16%	↓ 29%		(268)
Broiler	42 days	35 ± 2°C	↓ 15.2%	↓ 19.2%		(225)
Broiler	35 days	TN: 20°C HS: 27.8 °C	↓ 5.0%	↓ 17.9%		(271)

BW, body weight; HS, heat stress; K^+^, potassium ion; N/A, not available; Na^+^, sodium ion; pCO_2_, partial pressure of carbon dioxide; RH, relative humidity; TN, thermoneutral.

Literature shows that HS adversely impacts fat metabolism, impedes muscle growth, and detrimentally affects meat quality and chemical profile (55–58). According to Lu et al. (59), extended periods of heat stress adversely impact fat accumulation and meat quality. This was primarily attributed to electrolyte imbalance and lipid peroxidation. Dai et al. (56) and Imik et al. (60) stated that HS reduces broiler meat’s chemical composition and quality. Zhang et al. (61) reported that chronic HS reduced the breast muscle proportion and increased the thigh muscle in broilers. In the same study, birds exposed to HS had lower protein content and higher fat deposition in their meat. Broilers under HS usually exhibit lighter carcasses, reduced breast muscle, and increased abdominal and subcutaneous fat. Muscle protein accretion is hindered by heat-induced proteolysis and reduced muscle protein synthesis, while at the same time, lipogenic enzymes increase, depositing extra fat in tissues (33). This leads to poorer meat quality, characterized by paler, softer meat with lower water-holding capacity and pH and higher drip loss during storage (62). Therefore, in heat-stressed flocks, meat characteristics, including the meat color, texture, juiciness, and flavor, are often adversely affected.

Chronic heat stress induced pale, soft, exudative-like meat traits in broilers due to protein denaturation and muscle fiber shrinkage (61). Additionally, chronic heat exposure can lead to muscle fiber atrophy in broilers, reductions in muscle fiber diameter, and changes in muscle histology, which have been reported to correlate with reduced breast meat yields (61). Unlike chronic HS, acute HS has less pronounced effects on meat quality, but there is evidence that acute HS alters blood chemistry as an indication of skeletal muscle cell injury (63). Acute HS induces rapid metabolic shifts, including increased oxidative stress and increased glycogen utilization, which can temporarily impair muscle function. Under acute HS, the muscle production of lactate increases, leading to a decrease in pH and subsequently decreasing breast meat quality (64). High environmental temperatures can adversely affect ribosomal capacity by altering ribosomal gene transcription, which reduces protein synthesis and increases protein degradation (61). Increased fat accumulation may result from a reduced basal metabolism combined and physical activity (65).

Research has shown that HS negatively affects nutrient digestibility in broilers (66), laying hens (67), and quails (68). In broilers exposed to either continuous or cyclic HS, energy and nutrient digestibility were affected by HS, with dry matter and protein digestibility decreasing by 3.9 and 9.7%, respectively (66). The digestibility of essential amino acids is impacted by HS, causing a slight decrease noted for threonine, alanine, methionine, isoleucine, and leucine, and more significant reductions were observed in male birds (69). The digestibility of neutral detergent fiber was reduced in laying hens (67).

The mechanisms through which HS impairs nutrient digestibility and utilization are complex, and numerous studies have sought to clarify them. Evidence indicates that HS significantly downregulates the expression and activity of key digestive enzymes, including amylase, chymotrypsin, lipase, and maltase (70, 71). Additionally, the oxidative stress from HS caused intestinal barrier dysfunction, adversely affecting nutrient absorption efficiency (33). Heat stress alters the expression of genes coding for several macronutrient transporters. Several studies have documented that the occurrence of high temperatures over a prolonged duration results in a reduction in the expression of the glucose transporters, GLUT-2 and SGLT-1 (71–74). On the other hand, GLUT-5 expression rises, aiding in fructose transport (73). Conversely, there is an upregulation of GLUT-5, which is responsible for facilitating the transport of fructose (73).

### 3.2 Impact of heat stress on laying performance

Heat stress stimulates the avian HPA axis to elevate hormonal levels, including adrenocorticotropic, catecholamines, and corticosterone (75). This hormonal surge negatively impacts FI and metabolism, ultimately reducing overall performance (14). Kumar et al. (76) reported that when laying birds were exposed to HS, egg production, egg weight, BW, BWG, and daily FI were decreased by 4.99–57, 2.78–14.3, 3.74–32.6, 11–50, and 16.09–46.33%, respectively. Mack et al. (41) reported that HS reduced egg weight (13.5%) and eggshell thickness (10.5%) in White Leghorn hens. The quality of internal egg components may also be adversely affected, as producers frequently report diminished albumen quality, as evidenced by a reduction in albumen height and decreased Haugh unit scores, during periods of elevated temperatures. This decline in quality is potentially attributable to diminished FI, which can result in nutrient deficiency, as well as oxidative stress that adversely impacts yolk pigmentation and lipid stability (77).

High temperatures adversely affect FI in breeders and laying hens by decreasing FI and productive efficiency throughout the egg production cycle. While investigating the effect of HS on the ovarian function of laying hens, Rozenboim et al. (78) reported that exposing White Leghorn laying hens to heat stress (42 ± 3°C) for two days resulted in a 20% reduction in egg production. In the same study, the hens’ egg weight declined after a day of exposure to HS. Also, Buranawit et al. (79) reported that chronic HS reduces laying hens’ egg production and weight. Likewise, HS disrupts the hen’s endocrine balance, which is necessary for reproduction. Lowered estrogen levels lead to fewer mature ovarian follicles and contribute to the decline in egg production (80). Preferably, laying hens should attain sexual maturity at a weight marginally above the standard weight to induce increased food consumption, improved egg weight production, and enhanced consistency in egg production. Birds kept at 35°C have a 20–30% reduction in BW at sexual maturity when compared to hens raised at 21°C (17). Consequently, the overall egg production and quality are negatively impacted (81).

The impact of environmental temperature becomes important when laying hens attain sexual maturity. For every 1°C increase above the optimal temperature, the average feed consumed decreases by 1.6%, while the energy expenditure (metabolic energy use) increases by 2.3% (17). When temperatures exceed 30°C, both FI and egg production decrease, leading to a decline in saleable eggs (82). Notably, feed consumption was observed to decrease by 50% when temperatures increased from 21 to 38°C (17). Increasing the temperature-humidity index (THI) from 25 to 29 (29-34 °C; 34-58% relative humidity) led to a 25% reduction in total egg production (83). This decline in egg production and weight due to HS can be addressed by enhancing the nutritional content of poultry diets; however, this method does not mitigate the effects of HS on eggshell quality (17).

The THI serves as a critical environmental indicator, often used for predicting production losses that arise from livestock exposure to humid and hot climate (84). For laying hens, Zulovich and DeShazer (85) set different levels of THI: comfort (THI < 70), alert (70 < THI < 75), danger (76 < THI < 81), and emergency (THI > 81). As THI levels increase, hens experience reduced FI and lower egg production (86), and deteriorated egg quality parameters (86). Notably, higher THI levels (THI = 85) led to decreased eggshell thickness and strength, egg yolk color intensity, and Haugh unit (86).

### 3.3 Impact of heat stress on reproductive function

Exposure to HS has a more pronounced impact on infertility in male breeders than on female breeders (87). Seminal characteristics, including semen production, quality, motility, and sperm metabolism, are modulated by temperature, pH, and ion concentration. When these factors are altered, they may ultimately result in infertility and the production of low-quality spermatozoa (88–90). When exposed to temperatures exceeding the thermoneutral zone, poultry species, including Japanese quail (91, 92) and broiler chicken (93), suffered from testicular abnormalities and dysfunction. These abnormalities adversely affected seminal parameters, including testicular spermatogenic cell counts, Bax (an apoptotic marker) immunopositive staining, levels of testicular Bcl-2 (an anti-apoptotic marker), the Bax/Bcl-2 ratio, and the quantity of androgen receptors, which were associated with an increase in testicular lipid peroxidation. Testicular abnormalities affect the germ cells responsible for spermatogenesis. Consequently, heat-stressed male birds exhibit reduced sperm production and impaired sperm quality, characterized by decreased motility and viability (94). Also. HS can cause testicular atrophy, shrinking the size of the testes and leading to hormonal imbalances, including reduced levels of testosterone, which further hinders reproductive function (94, 95). Ameen et al. (96) reported that HS reduced the reproductive efficiency of male cockerel breeds as semen quality and quantity were affected. Additionally, inseminating hens with semen obtained from roosters exposed to HS resulted in a higher percentage of unfertilized eggs, which is attributed to decreased sperm-egg penetration. This corroborates the report of McDaniel et al. (97), which stated that the rate of in-vivo sperm penetration into eggs was 48% lower at 27°C than at 21°C in broilers. Obidi et al. (98) reported that high temperatures promote testicular growth by increasing semen volume and concentration in the early phase of development. However, excessive heat later suppresses reproductive capacity in poultry.

Several studies have reported that exposing breeder and laying hens to excessive heat adversely affects the species’ ovulation rate, resulting in reduced fertility (89, 99), reproductive performance (98, 100, 101), and hatchability (102, 103). Heat stress lowers estrogen and progesterone levels, resulting in impaired ovarian follicular development (fewer mature ovarian follicles) and contributing to reduced egg production (80). Additionally, hens exposed to high temperatures experience infertility issues, including reduced follicular and oocyte development, and lower yolk maturation rate (104, 105). Ayo et al. (101) suggested that this effect could be due to reduced secretion of luteinizing hormone (LH), follicle-stimulating hormone, gonadotropin-releasing hormone, and alterations in antioxidant levels, fatty acid composition, and heat shock proteins (HSPs). Laying hens exposed to acute HS experienced disrupted reproductive function regulation, with reduced circulating LH levels due to reduced hypothalamic function (105). Breeder hens inseminated in the afternoon had lower fertility and hatchability rates than those inseminated in the morning (98), suggesting a potential temporal influence on reproductive success in avian species. Heat stress induces oxidative damage to the small yellow follicles, ovaries, and oviducts of ducks, laying hens, and quails, resulting in reduced relative reproductive organ weights and a decrease in the number of large follicles (76). Consequently, it leads to lower egg production, and in extreme instances, it can cause infertility (106–109).

### 3.4 Impact of heat stress on gut health

The gut is essential for digesting and absorbing nutrients, maintaining electrolyte balance, and supporting immune system development in living organisms (110). The gut ecosystem is inhabited by various microorganisms, including archaea, bacteria, and protozoa. Several factors, including dietary composition, environmental stressors, and temperature fluctuations, can disrupt this ecosystem (111, 112). Gastrointestinal health is adversely affected following HS, leading to reduced nutrient absorption, immune response dysfunction, and compromised gut epithelium integrity (21). In a thermoneutral environment, the gastrointestinal tract primarily absorbs nutrients through transcellular transport, a process in which molecules move across the cell membranes of intestinal epithelial cells. This mechanism is facilitated by various specific receptors and transporters located on these cells’ apical (lumen-facing) and basolateral (blood-facing) membranes. For instance, glucose and amino acids are absorbed transcellularly through sodium-dependent transporters like SGLT-1 and various amino acid transporters, while fatty acids and monoglycerides enter the enterocytes either by diffusion or through fatty acid transport proteins. While transcellular transport is dominant, a smaller portion of nutrient absorption can also occur via the paracellular pathway, which involves movement between the cells through tight junctions (TJ). This pathway is largely passive and driven by concentration gradients (113). Examples of nutrients that can be absorbed paracellularly include small ions such as sodium (Na^+^), chloride (Cl^−^), and water-soluble minerals like magnesium (Mg^2+^). The epithelial cells of the gut are interconnected through intercellular junction complexes to form intestinal barriers, stabilizing the epithelial barrier’s integrity (114, 115).

The junction complexes of gut epithelial cells include TJ, adherent junctions (AJ), desmosomes, and gap junctions (GJ) (116). The AJ is located beneath the TJ and contains cadherin and *β*-catenin as an extensive attachment to a ring of peri-junctional actin filaments and is involved in intracellular communication. Both TJ and AJ connect to the actin cytoskeleton (117, 118). Desmosomes are found beneath the AJ and are associated with keratin filaments. Desmosomes facilitate cell adhesion and protect the alimentary epithelia from shear-induced damage, while GJs are key in intracellular signaling (119). The cytoskeleton is a complex of proteins that supports the structure of all eukaryotic cells, and any damage to it can lead to an impairment of intestinal integrity (120).

**Figure 4:**
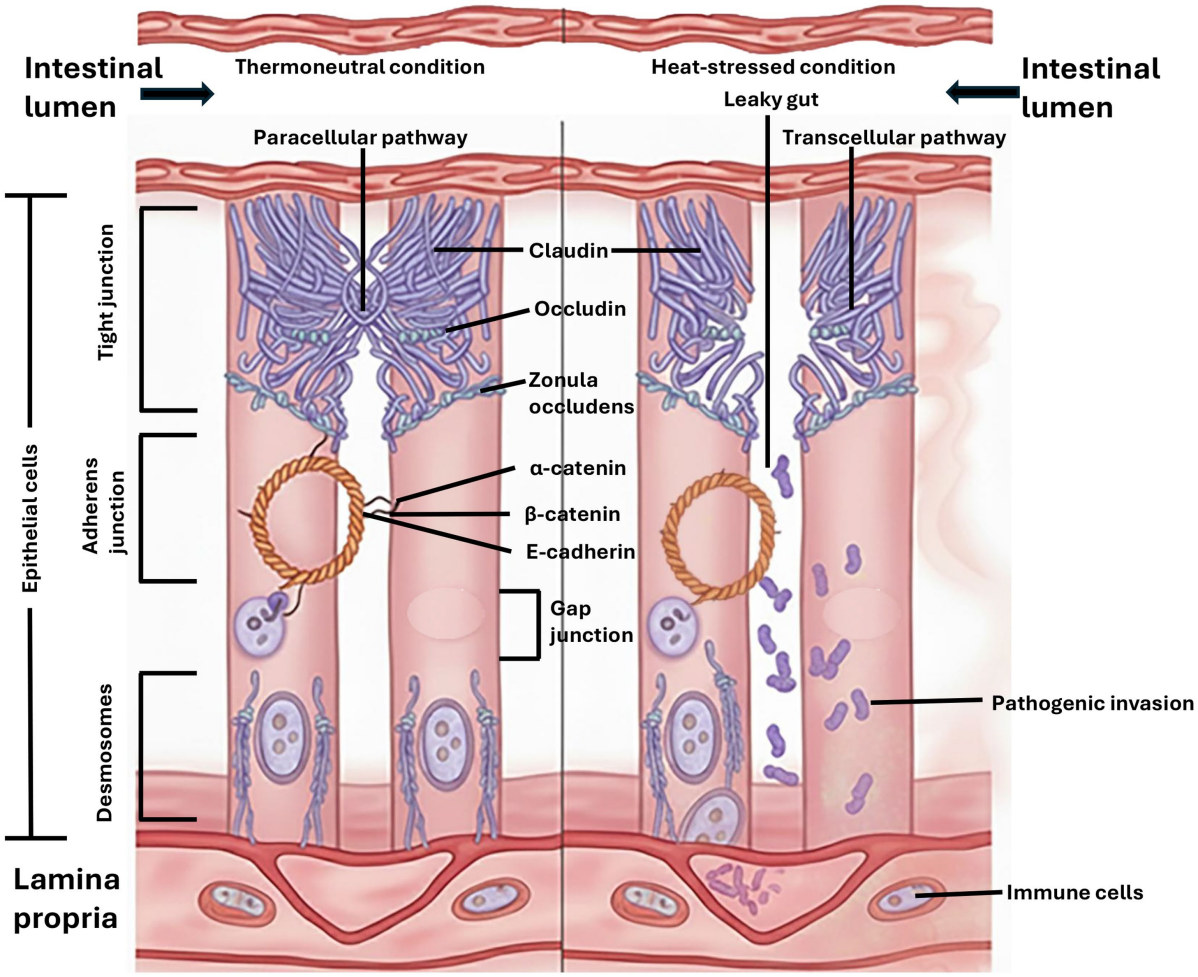
Mechanism of heat stress-induced intestinal permeability. Under thermoneutral conditions, the tight and adherens junctions form an intact barrier. Under heat stress, oxidative damage disrupts these junctions, widening the paracellular pathway and leading to the invasion of pathogenic bacteria and endotoxins into the lamina propria, triggering inflammation.

The TJ facilitates the transportation of several materials and is controlled by signaling pathways (21). Claudin genes encode the protein “claudin” that forms strands at the TJ barrier, conferring cell–cell adhesion. Claudins are barrier-forming proteins responsible for regulating paracellular permeability through the pore pathway. Occludin modulates the paracellular leak pathway, and its phosphorylation results in the disruption of the TJ. Therefore, the concentration of claudin and occludin is vital for the regulation of TJ barrier functions (120). The TJ barrier is compromised in high-temperature environmental conditions, allowing luminal contents to enter the bloodstream (Figure 4). When this occurs, the gut becomes leaky and results in chronic systemic inflammation, which impairs the immune competence of birds (119).

Several studies have indicated that HS negatively impacts nutrient digestion and absorption while enhancing the intestine’s sensitivity to pathogens (107, 108, 121–127). Heat stress alters intestinal morphology by causing abrasions in the duodenum, jejunum, and ileum (128). Due to increased reactive oxygen species levels, which heighten lipid peroxidation in the intestinal cell walls and pancreas, HS reduces the production of digestive enzymes (128). Additionally, there is a proliferation of pathogenic bacteria and a decline in beneficial microbiota, which compromises gut (121). This results in reduced meat and egg yields, compromised immune response, and hampered overall production performance.

### 3.5 Impact of heat stress on acid–base balance and electrolytes

**Figure 5:**
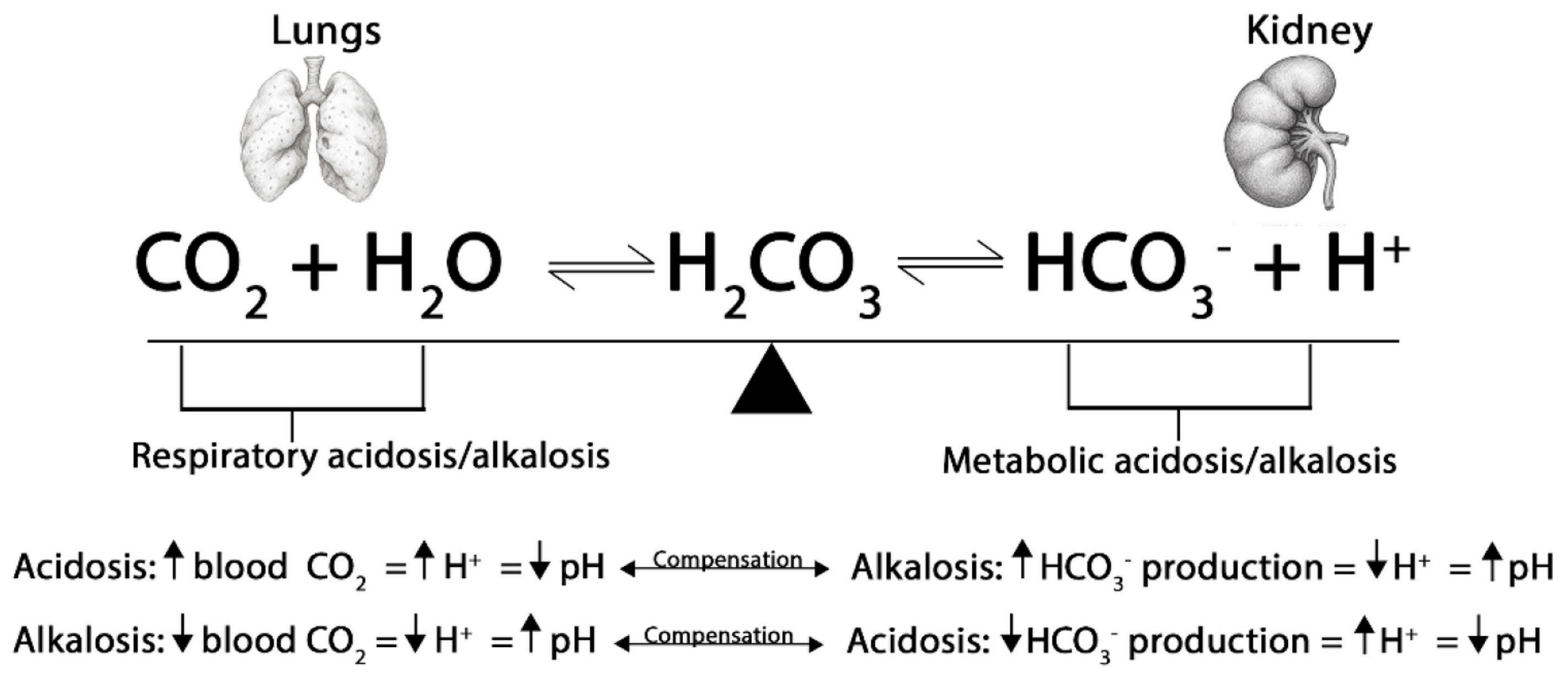
Respiratory and metabolic acidosis and alkalosis. The process of breathing leads to the production of CO_2_, which subsequently enters the bloodstream. Blood, primarily composed of water, facilitates a chemical reaction when carbon dioxide mixes with it, resulting in the formation of H_2_CO_3_. Carbonic acid is unstable and dissociates into HCO_3_^−^ and H^+^. Thus, the lungs regulate blood pH by altering the PaCO_2_, while the kidneys regulate blood pH by altering the concentration of HCO_3_^−^. When the pH is too acidic, the kidneys absorb HCO_3_^−^, and when it is too alkaline, they excrete HCO_3_^−^.

At normal temperatures, the kidneys and lungs function together to maintain acid–base equilibrium in the blood at a pH between 7.35–7.45 (Figure 5). Birds maintain pH via the bicarbonate buffer system and respiratory exchange of carbon dioxide (CO_2_). The hydration of CO_2_ generates hydrogen ion (H^+^) and bicarbonate (HCO_3_^−^), with hemoglobin and other buffers capturing H^+^ as CO_2_ is exhaled. This interaction of H^+^ with HCO_3_^−^ to synthesize carbonic acid (H_2_CO_3_), thereby regulating the blood pH. Carbonic anhydrase converts H_2_CO_3_ into CO_2_ and H_2_O. The lungs expel the resulting CO_2_, while the kidneys excrete H^+^ ions along with HCO_3_^−^ (27).

During HS, a cascade of physiological changes occurs, starting with panting (respiratory rate increases from 25 breaths/min to 260 breaths/min), which increases CO_2_ exhalation relative to its cellular production, thereby altering the standard bicarbonate buffer system in the blood. The decreasing CO_2_ concentration in the blood results in a lower concentration of H_2_CO_3_ and H^+^. Conversely, the levels of HCO_3_^−^ rise, leading to an increase in blood pH or respiratory alkalosis (28). Hyperventilation causes the dehydration of H_2_CO_3_ to CO_2_ and water (H_2_O), thereby depleting H^+^ and increasing pH (32). As a mechanism to normalize the blood pH, birds will begin to excrete higher levels of HCO_3_^−^ and retain H^+^ in the kidney. Contrarily, the body may also experience a decrease in plasma HCO_3_^−^ levels and a lower blood pH due to the increased production of lactic acid in the muscles, causing metabolic acidosis. This can occur when the body is not receiving enough O_2_ to meet the demands of the muscles during periods of high activity, such as panting. This altered H^+^ disturbs acid–base homeostasis, leading to respiratory alkalosis and metabolic acidosis, which are linked to reduced performance in poultry (129).

Respiratory acidosis involves a primary increase in the arterial partial pressure of carbon dioxide (PaCO_2_) and a consequent decrease in blood pH. Hypoventilation prevents adequate CO_2_ exhalation, leading to H_2_CO_3_ accumulation and an elevation of H^+^ (130). In acute respiratory acidosis, pH drops rapidly, and birds compensate for this by increasing HCO_3_^−^ reabsorption and H^+^ excretion (renal compensation). In contrast, metabolic alkalosis results from elevated HCO_3_^−^ levels, causing an increase in blood pH. This condition can occur due to a net loss of H^+^ or an accumulation of HCO_3_^−^ (131). Respiratory compensation involves hypoventilation, which retains CO_2_, and the kidneys’ retention of H^+^. Metabolic alkalosis is often associated with hypokalemia because H^+^ exits cells in exchange for potassium ion (K^+^), resulting in an elevated blood pH, while the kidneys reabsorb Na^+^ alongside HCO_3_^−^.

The production of calcium carbonate in the shell gland is essential for eggshell formation and depends on adequate HCO_3_^−^ level in laying hens (129, 132). Alkalosis reduces the blood ionized calcium concentration, which impairs eggshell mineralization in laying hens and contributes to poor growth in broilers (133). Consequently, acute HS can jeopardize calcium-dependent processes, such as eggshell formation, along with overall homeostasis. Chronic HS worsens these problems since initial rapid, shallow panting can evolve into deeper panting, known as “thermal hyperpnea,” as birds attempt to cope with prolonged heat (134). Frank and Burger (135) and Balnave and Muheereza (136) reported that lower bicarbonate levels in the shell gland lumen negatively influence eggshell quality. Likewise, Miller and Sunde (137) stated that an abrupt increase in air temperature negatively affects eggshell quality within just one oviposition cycle, highlighting that respiratory alkalosis in hens sharply reduces blood ionized calcium levels (133).

Besides altering the acid–base balance, HS also alters electrolyte concentrations, particularly Na^+^, K^+^, and Cl^−^, which are crucial for intracellular and extracellular fluid homeostasis (138). Osmoregulation is preserved through tight control of intracellular K^+^ and extracellular Na^+^ and Cl^−^. In broiler chickens, the recommended dietary electrolyte balance (DEB; total of Na^+^ + K^+^ − Cl^−^, mEq/kg) is around 250 mEq/kg feed to support acid–base equilibrium (139), although this may vary with ambient temperature. To maintain electrolyte homeostasis in body fluids, the body increases the excretion of K^+^ and Na^+^ in feces and urine, while the Cl^−^ concentration in the blood increases. According to Borges et al. (140), HS reduced plasma Na^+^, K^+^, and PaCO_2_ concentration, likely due to hemodilution from elevated water level consumption. Similarly, in heat-stressed birds, elevated temperatures decreased plasma Ca, Na, inorganic P, and Mg (141).

## 4 Nutritional supplements used in poultry for alleviating heat stress

Heat stress adversely impacts poultry growth, nutrient digestibility, immunity, and overall welfare, leading to poor performance, increased mortality rates, immune dysfunction, and economic losses (142, 143). Several strategies and management practices, including housing, physical cooling measures (134), ventilation, thermal manipulation, *in ovo* feeding, and breeding for heat-resistant related genes (144) have been implemented to alleviate HS in poultry. Yet, the effect of HS persists in the poultry industry. This has prompted the search for alternative strategies and nutritional manipulations to address this challenge (145, 146). The potential of several nutritional supplements, including vitamins, minerals, fat, and amino acids, has been investigated.

### 4.1 Vitamins

Researchers have extensively studied the role of vitamins in reducing the negative impacts of HS (147, 148), with vitamins C and E identified as having the most significant influence (34). Vitamins C and E are incorporated into poultry diets because of their anti-stress and antioxidant effects, and their production decreases during HS. Supplementing birds’ diet with vitamins A, C, and E improved egg production, hatchability, and fertility while also reducing egg breakages and mortality in laying hens in hot environments (149). Chung et al. (150) and Abd El-Gawad et al. (147) reported that supplementing vitamins C and E into the diet of heat-stressed broilers and quails improved eggshell quality, increased FI, and boosted BW.

#### 4.1.1 Vitamin C

Vitamin C, or ascorbic acid, is a water-soluble antioxidant that protects cells from oxidative damage and is vital for stress tolerance, including HS in poultry (104). High temperature increases the birds’ demand for vitamin C. During hot weather, the tissues actively absorb and utilize vitamin C, leading to insufficient availability of plasma ascorbic acid and increased demand for vitamin C (34). Chickens naturally produce ascorbic acid in the kidney from glucose (151), which meets their normal physiological needs under typical conditions (152). During HS, the demand for ascorbic acid significantly increases because the endogenous synthesis of vitamin C fails to meet the birds’ physiological requirements. Ascorbic acid is an essential nutrient that is critical in maintaining efficient homeostasis. Chickens need vitamin C for the effective metabolism of amino acids and minerals, along with synthesizing hormones that help manage stress. It promotes leukocyte activity and contributes to antibody synthesis (104). As environmental temperatures rise, blood ascorbic acid levels decline; thus, supplemental ascorbic acid could help lessen the impact of HS in poultry.

Layers are susceptible to HS due to the higher metabolic heat produced during egg formation, ovulation, and oviposition (153). Ascorbic acid is crucial to several essential functions, like the release of adrenaline and corticosterone, the biosynthesis of 1,25-dihydroxy vitamin D and collagen, and the enhancement of calcium metabolism (154). Supplementing vitamin C enhances heat-stressed birds’ performance by boosting feed consumption and nutrient uptake. Asensio et al. (155) demonstrated that the inclusion of ascorbic acid in broiler diets significantly improved carcass weight and protein content, alongside a notable reduction in carcass fat. Supplementing diets with 50 and 100 mg/kg of vitamin C increases the fertility and hen-day egg production of broiler breeders (156). According to Attia et al. (157), supplementing 200 mg/kg vitamin C in the diets of heat-stressed laying hens improved FI, egg production, and egg quality. Furthermore, including 200–400 mg of ascorbic acid/kg of laying hens’ diet improved the number of produced eggs, survival rate, and FI (158). Also, Ahmed et al. (159) found that incorporating vitamin C into laying hens’ diets at 1,000 and 1,200 ppm/L of water optimized their egg production rate, egg weight, egg mass, livability, and FCR during HS.

#### 4.1.2 Vitamin E

Vitamin E consists of various fat-soluble compounds known for their antioxidant benefits, including tocopherols and tocotrienols. Supplementing vitamin E in poultry diets is essential in poultry nutrition because birds do not have the capability to synthesize it (157). Including vitamin E in the diet during stressful periods enhances the immune response in poultry. During stressful conditions, vitamin E is a crucial natural antioxidant that reduces physiological stress induced by corticosterone and catecholamines. During HS, the hormonal levels of catecholamine and corticosterone increase, leading to the commencement of peroxidation of lipid in cell membranes (160). Vitamin E can serve as the primary defense against lipid damage and peroxidation of cells and tissues and effectively neutralizes free radicals (157, 161).

Heat stress induces oxidative stress, which impairs immune functions, but antioxidants can avert this impairment. Immune cells are rich in polyunsaturated fatty acids, making them highly susceptible to oxidative damage (162). Due to their lipophilic nature, antioxidants like vitamin E and carotenoids are efficacious in neutralizing damaging reactive species in lipid-rich membrane environments (163). According to Shakeri et al. (164), vitamin E is important in immune responsiveness by protecting plasma cells, lymphocytes, and macrophages from oxidative stress and likewise improves their viability and proliferation function. The exposure of Japanese quails to 34°C for 8 h/day, followed by 22°C for 32 days, led to a 29% decrease in blood tocopherol levels compared to the control group under normal temperature (165). This finding suggests that prolonged HS results in a reduction of antioxidant components.

Supplementing broiler chickens’ diet with vitamin E at 250 mg/kg effectively mitigates the severity of HS, potentially leading to improved bird performance and superior meat quality (166). Laying hens had improved feed utilization, egg production, and immunological response when vitamin E was supplemented into their diets at 125–250 mg/kg (164). Heat stress elevated malondialdehyde (MDA) levels in the blood and liver, whereas vitamin E supplementation reduced MDA formation, preventing lipid peroxidation and cell damage (167), to improve chicken performance. In high ambient temperature conditions, dietary vitamin E supplementation at 250 mg/kg increased the activity of glutathione peroxidase (GSH-Px) and reduced the levels of HSP60 in broilers (168). Likewise, supplementing 200 mg vitamin E/kg diet enhances the antioxidant defenses in chicken semen subjected to HS. This enhancement is due to a reduction in lipid peroxidation, improvement in GSH-Px activity, and an increase in total antioxidant capacity as measured by levels of MDA (161). Additionally, it enhances the fatty acid profile, feed consumption and efficiency, egg yield and quality, and oxidative stability of thigh muscle in broilers and quail raised under HS (148, 149, 169).

##### 4.1.2.1 Effects of vitamins on growth and nutrient digestibility/utilization

Vitamins play significant roles in improving growth and nutrient utilization in heat-stressed birds (Table 3). Under cyclic HS (36 ± 2°C), broilers fed diets supplemented with vitamin E (100 mg/kg), vitamin C (200 mg/kg), and probiotics enhanced BWG and decreased the FCR of broiler chickens (170). Also, McKee et al. (171) reported that vitamin C (150 mg/kg in diet plus 400 mg/L in water) mitigated the reductions in cockerels’ weight gain, FI, and gain:feed ratio caused by heat exposure. Similarly, dietary inclusion of 100 mg vitamin E/kg enhanced feed utilization in broilers under heat stress, as evidenced by a significant decrease in FCR (172). These observed benefits could be associated with the antioxidant properties of the vitamins. By mitigating oxidative damage and gut permeability induced by HS, they stabilize the cell membranes and improve immune response, improving nutrient absorption and allowing birds to partition more energy toward growth.

**Table 3:** Effects of vitamins and mineral supplements on heat-stressed birds.

Avian species	Heat stress condition	Intervention	Dosage	Effect summary	References
Laying hens (Bovans Brown; 30 weeks old)	30–37°C each day from 30–38 weeks of age	Organic mineral mixture (Mn, Zn, Cu)	1 g/kg	Enhanced reproductive performance, immune response, and egg quality	(276)
Broilers (Hubbard and Cobb; 1-day-old)	29–36°C; 52–65% RH during weeks 3–6	Vitamin E	250 mg/kg	Positive effects on antioxidant and immune responses	(277)
Broiler chicks (Cobb 500; 1-day-old)	Chronic heat stress (36 ± 2°C; 75–85% RH for 7 h/day during 25–42 days of age)	Vitamin E Vitamin C Probiotics (*Saccharomyces cerevisiae* and *Lactobacillus acidophilus*)	100 mg /kg 200 mg /kg 2 g/kg	Reduced some adverse effects of heat stress, with the combination of all three being most effective Probiotic + Vitamin E + Vitamin C increased BWG and decreased FCR of the heat-stressed broilers by 10.5 and 8.4%, respectively	(170)
Broilers (Cobb strain; 1-day-old)	27–37°C; 85–94% RH for 5 weeks	Potassium chloride	0.00, 0.25, 0.50, 0.75 and 1.00% (w/v) in drinking water	Improved performance and physiological responses	(278)
Broilers (Cobb 400; 1-day-old)	35°C for 21 days	Zinc	40 mg/kg	Improved growth and reduced FCR	(194)
Broilers (Ross 308; 1-day-old)	35 ± 1°C for 15–30 days of age	Nano-selenium Sodium selenium	0.3 mg/kg 0.3 mg/kg	Improved BWG (14%), antioxidative status, immune response, and reduced FCR (14%) Nano-selenium increased glutathione peroxidase and cytokines (IL-2 and IL-6) mRNA expression	(279)
Laying hens (Mandarah strain; 32 weeks old)	Chronic heat stress (38 ± 1°C; 55–65% RH for 4 h/day on 3 consecutive days/week for 32–48 weeks)	Betaine Vitamin C Vitamin E	1,000 mg/kg 200 mg/kg 150 mg/kg	200 mg vitamin C/kg diet and 1,000 mg betaine/kg diet enhanced laying performance All the feed additives, alone or in combination, alleviated the effect of heat stress on feed efficiency, with a reduction in FCR Vitamin C increased the heat-stressed hen’s laying rate by 8% Betaine increased the egg mass of the heat-stressed hens by 10% Vitamin E + betaine increased the protein digestibility of the heat-stressed hens by 3% Betaine increased the ovary weight of the heat-stressed hens by 9%	(157)
Broilers (Cobb 500; 1-day-old)	Cyclic heat stress (23.9–37°C; ≥ 55% RH for 8 h/day during 4–7 weeks of age)	Vitamin E Selenium	0, 125, and 250 mg/kg 0, 0.5, and 1 mg/kg	Improved feed efficiency and immune response Vitamin E decreased the heterophil:lymphocyte	(206)
Cockerels (Egyptian local cross, Mandarah); 40 weeks old	33–36°C; 60–70% RH for 8 weeks	Vitamin E Organic Selenium	200 mg/kg 0.3 mg/kg	The synergistic effects of vitamin E and Se improved semen quality, reduced lipid peroxidation, and enhanced antioxidative status	(161)

BWG, Body weight gain; FCR, Feed conversion ratio; h, Hour(s); IL-2-, Interleukin-2; IL-6-, Interleukin-6; RH, Relative humidity; ROS, reactive oxygen species; Se, Selenium.

In Japanese quails, Sahin and Kucuk (169, 173) demonstrated that dietary vitamin C (100–200 mg/kg) and vitamin E (125–500 mg/kg) increase final BW and feed efficiency. Also, the digestibility coefficients for dry matter, organic matter, and crude protein were improved. Combining vitamin C with folic acid increases mineral retention in Japanese quails. When vitamin E was combined with selenium (Se), the digestibility parameters, especially dry matter, were improved (173). In several studies, the combined use of vitamins with or without Se yielded synergistic effects on performance and nutrient utilization. Sahin and Kucuk (173) reported a significant interaction for final BW and feed efficiency between vitamin E and Se supplementation in Japanese quails exposed to HS (34°C). This suggests that strategic combinations of vitamins in the presence or absence of Se may be more effective in supporting growth performance and nutrient utilization in heat-stressed poultry than single vitamin supplementation.

##### 4.1.2.2 Effect of vitamins on skeletal health

Heat stress induces oxidative stress that directly compromises the gut barrier function, leading to a massive reduction in the absorption of bone minerals like calcium and phosphorus (174). Vitamin supplementation could enhance the skeletal health of heat-stressed poultry by mitigating gut damage and maintaining efficient mineral absorption. Calik et al. (175) demonstrated that supplementing vitamin E and Se into the diets of broilers exposed to cyclic HS (35°C for 4 h/day) resulted in an enhanced bone mineral content (BMC) and bone mineral density (BMD). Due to their antioxidant properties, these additives protected the intestinal epithelium from this oxidative damage, maintaining the gut integrity. This facilitates the transport of calcium and phosphorus across the mucosa for utilization, thereby enhancing BMC and BMD. Similarly, Marques et al. (176) investigated the efficacy of 25-hydroxycholecalciferol (25-OH-D_3_) as an alternative to conventional vitamin D_3_ in broilers exposed to HS (31.1–32.9°C). Their findings indicated that 25-OH-D_3_ facilitated improved calcium and phosphorus deposition in bones, ensuring skeletal integrity without compromising overall performance, even when dietary Ca levels were reduced.

Contrarily, the observations of Mosleh et al. (177) regarding ascorbic acid supplementation indicated a limited impact on bone mineralization. While vitamin C alleviated oxidative stress in broilers exposed to chronic HS (39°C for 8 h/day over several durations), it did not significantly enhance BMD or other bone characteristics. This suggests that vitamin C alone may be insufficient to prevent heat-induced skeletal deterioration. However, Sahin et al. (178) found that combining 25-OH-D_3_ with soy isoflavones in quails exposed to 34°C for 8 h/day led to marked improvements in BMD, tibia ash content, and serum calcium and phosphorus concentrations. This suggests that a synergistic approach involving vitamin D metabolites and phytoestrogens may be particularly beneficial for maintaining bone health under HS conditions.

### 4.2 Minerals

Heat stress triggers several responses, including physiological and behavioral reactions in poultry. In response to excessive heat, birds reduce FI, which leads to insufficient mineral intake to meet their need. Therefore, heat-stressed birds are deficient in minerals, making the supplementation of certain minerals a priority. Minerals provide support for several biological and cellular functions, facilitating growth, enhancing nutrient utilization, strengthening immune responses, mitigating oxidative stress, and supporting the overall productivity of birds subjected to HS (179, 180). Heat stress in poultry causes increased mineral excretion, resulting in acid–base imbalances and respiratory alkalosis. This issue could be addressed with appropriate mineral supplementation at various production stages (181). This review focuses on zinc (Zn), Se, and chromium (Cr) supplementation due to their antioxidant properties, gut barrier integrity maintenance, proper immune functions, and glucose metabolism and energy utilization, which are negatively affected by HS.

#### 4.2.1 Zinc

Zinc is an indispensable trace element that is important for homeostasis and acts as a cofactor for several enzymes involved in many biological and life processes, like skeletal development, antioxidant defense system, and immune response (106, 182). Zinc supplementation is vital because the body cannot store it. Furthermore, zinc preserves the integrity of the DNA, so its deficiency negatively impacts the DNA by reducing the effectiveness of zinc-dependent proteins (183). During HS, Zn protects the cells by enhancing antioxidant status, scavenging ROS, and attenuating heat shock responses. Zinc is vital for metallothionein synthesis, a free radical scavenger (182).

As an important constituent of antioxidant enzymes, like glutathione (GSH), superoxide dismutase (SOD), and glutathione S-transferase, Zn helps to mitigate free radical formation. Additionally, Zn is an element of carbonic anhydrase, which is responsible for catalyzing carbonate synthesis, which is crucial for eggshell mineralization (184). By negatively regulating the nuclear factor kappa-light-chain enhancer of activated B cells (NF-κB) signaling pathway, it helps to suppress inflammation responses. The import of Zn ions into cells via the Zn transporter protein family 8 (ZIP8) may inhibit the Ikappa-B kinase complex to downregulate NF-κB during inflammatory responses (185).

Zinc is often included in the diets of breeding birds to enhance reproductive function. Zinc deficiency reduces semen quality (about 10% decrease in sperm motility), lowers egg production, and leads to abnormal embryonic development and poorly performing offspring (186). Zhu et al. (187) found that maternal HS at 32°C during the age of 33 to 42 weeks negatively impacted both hatching performance and embryonic development in chickens; however, these negative effects could be mitigated by administering 110 mg Zn/kg of diets for 9 weeks. In heat-stressed broiler breeders, Zn supplementation at 110 mg/kg enhanced egg production and quality, improved antioxidant markers, and upregulated pancreatic and hepatic HSP expression (188). Turkey breeders that received supplemental Zn during the hot summer had increased egg production along with enhanced natural behaviors like feather cleaning and dustbathing (189). Also, corticosterone levels in plasma serve as a stress biomarker induction. Zinc supplementation lowered plasma corticosterone levels and enhanced egg production and BW in turkey breeders during the summer (190).

According to Long et al. (191), the supplementation of the organic form of zinc, zinc lactate, at varying doses of 40, 60, and 80 mg/kg in the diet enhanced broilers’ growth performance, intestinal morphology, antioxidant enzyme activity, immune response, and hepatic metallothionein. Organic Zn compounds might be more effective than inorganic Zn sources in reducing the adverse effects of HS in poultry. Zhang et al. (192) observed that the embryos from broiler breeders fed 80 mg Zn-Gly/kg of diet exhibited superior protection against HS-induced oxidative stress compared to those from breeders offered 80 mg ZnSO_4_/kg of diet. Additionally, Zn-lysine chelate significantly reduced *HSP70* and *HSP90* mRNA expression in heat-stressed broiler hepatocyte cultures compared to Zn oxide (193). In summer, broilers that received organic zinc supplementation at 40 mg/kg of feed had higher BWG, decreased lipid peroxide levels, and increased SOD enzyme activity (194).

Zinc performs several functions, including promoting metallothionein synthesis, regulating transition elements, and its connection with antioxidant vitamins like vitamins A and E. These functions might be responsible for its role in decreasing lipid peroxidation in birds receiving Zn supplements (195). Serving as a cofactor of the antioxidative enzyme, CuZn-SOD, Zn suppresses free radicals, impedes NADPH-dependent lipid peroxidation (196), and prevents lipid peroxidation by inhibiting the depletion of glutathione (197). Zinc can exert a direct antioxidant effect by competing and displacing transition metals (Fe, Cu) from binding sites, binding to the cell membrane, and decreasing free radical production (196, 198).

#### 4.2.2 Selenium

Selenium is a trace element essential for at least 25 selenoproteins, including antioxidant enzymes like thioredoxin reductases (TrxR) and GSH-Px (199, 200), which protect cells from oxidative damage and maintain redox homeostasis. In poultry nutrition, two forms of Se are used as feed additives: inorganic forms, like sodium selenite and selenite, and organic forms, like selenomethionine and Se yeast, with organic forms generally showing higher bioavailability (201). For proper antioxidative function, chickens require an adequate Se dosage of about 0.1–0.3 mg/kg diet (202). Due to its high toxicity, there is a threshold for the inclusion of Se in poultry diets (203). Therefore, both deficiency and excess of Se can impair poultry performance. Incorporating a high level of Se (3 mg/kg) into the chickens’ diet induced oxidative stress characterized by low SOD and catalase activities (204). Likewise, Se deficiency (0.03 mg/kg for 40 days) exacerbates the inflammatory status of heat-stressed broilers by increasing the ratio of M1-type inflammatory macrophages relative to M2-type anti-inflammatory cytokine-producing macrophages (205).

Selenium has an antioxidant potential and is used to alleviate HS in poultry (206, 207). In heat-stressed Japanese quail (34°C for 8 h/day), the supplementation of 0.3 mg of sodium selenite or selenomethionine per kilogram of diet enhanced egg production, egg quality, and the antioxidant profile of the birds (208). The most pronounced effects were observed in the quails that received selenomethionine supplementation. Also, supplementing broiler feed with Se (0.3 mg/kg) enhanced bird weight and reduced FCR during HS (209). The supplementation of inorganic sodium and sodium selenite, at 0.1 mg/kg and 0.2 mg/kg of feed, enhanced carcass quality and overall performance of heat-stressed quails (173). Selenium enhances the productivity and reproductive ability of laying hens (210). During thermal stress, incorporating selenized yeast into laying hens’ diets significantly enhanced various response variables, including egg weight, egg production rates, haugh unit, and eggshell strength (211). During periods of HS, the addition of Se (0.15 and 0.30 mg/kg of feed sodium selenite or selenomethionine) to the diets of laying quails led to an increase in FI, BW, egg production, and improved feed efficiency (208). Also, the study showed that supplementing both forms of Se increased the haugh units and eggshell weights.

In chickens, Se supplementation reduces the expression of the *HSP70* gene (204), whereas Se deficiency increases its expression in the spleen, thymus (212), and erythrocytes (213). Mahmoud and Edens (214) observed that the addition of Se yeast (0.2 mg/kg of diet) significantly modulates the *HSP7*0 response, enhances antioxidant status, and mitigates the incidence of enteric bacterial infections in heat-stressed animals. Kumbhar et al. (168) reported that supplementing 0.2 mg/kg of Se and 250 mg/kg of vitamin E reduced the expression of *HSP60*, HSP70, and *HSP90* in the breast muscle of heat-stressed chickens.

#### 4.2.3 Chromium

Chromium is an important trace element that is crucial in nutrient metabolism, including carbohydrates, proteins, and fats (215). It is an essential element of chromodulin and is required for insulin function (216). Heat stress leads to greater mobilization of Cr from body tissues, thereby raising nutritional needs (217). Supplementing poultry diets with Cr enhances feed efficiency (173), improves nutrient digestibility and transporter functions (218), and affects orexin and glucose transporters, as well as various biochemical parameters (219). In laying hens, incorporating 0.4–2.0 mg of Cr/kg of feed as chromium picolinate (CrPic) enhanced immunity, improved egg quality, and increased the haugh unit (220). Additionally, this supplementation reduced the level of blood glucose, cholesterol, and triglycerides in the hens (221). Furthermore, evidence supports that Cr supplementation helps alleviate HS-induced egg quality deterioration and metabolic imbalances in laying hens (222).

Research has also highlighted the effectiveness of organic Cr, such as CrPic and Cr-histidinate, in improving nutrient digestibility and metabolic stability in heat-stressed birds. Orhan et al. (218) found that laying hens subjected to 34°C for 8 h/d for 12 weeks, exhibited improved glucose, protein, and intestinal fatty acid transporters when supplemented with 200 μg CrPic or Cr-histidinate per kilogram of diet. Similarly, in a study involving 360 Japanese quails, dietary Cr-Pic supplementation (500 or 1,000 μg Cr/kg diet) improved growth performance, BWG, and biochemical regulation under HS conditions (223). Organic Cr exhibits superior efficacy in mitigating oxidative stress linked to physiological damage in animals subjected to HS when compared to inorganic Cr (224). Chromium methionine, an organic Cr, improved broilers’ cellular and humoral immune responses during HS (225). This enhanced effectiveness is likely attributable to the greater biodistribution, bioavailability, and absorptive characteristics of organic Cr (226).

Chromium has been extensively studied for its role in lessening the impact of HS in birds, primarily due to its antioxidant, anti-inflammatory, and metabolic regulatory functions. A primary mechanism through which chromium mitigates oxidative stress involves decreasing lipid peroxidation while enhancing total antioxidant capacity and GSH levels, which are essential for cellular defense against oxidative damage (227). Additionally, Cr exerts anti-inflammatory effects by inhibiting pro-inflammatory cytokines such as TNF-*α* and interleukin 6 (IL-6) which are often elevated under HS conditions (228). According to Sahin et al. (229), CrPic was more effective at reducing TNF-α, IL-6, and C-reactive protein (CRP) levels than CrCl_3_ in heat-stressed quails. Chromium anti-inflammatory role is likely associated with its capacity to increase the expression of *Nrf2* in heat-stressed birds (230). A study by Hamidi et al. (231) demonstrated that supplementing Cr nanoparticles or Cr-Pic (500, 1,000, or 1,500 mg/kg of diet) into broiler diets from 21 to 42 days of age enhanced immune responses by downregulating IFN-*γ*, which is crucial for ROS synthesis and inflammation under HS. Also, supplementing Cr-histidinate to heat-stressed quail diets (34°C for 8 h/day) can elevate hepatic inhibitor of NF-κB alpha (IκBα) levels while lowering NF-κB and influencing the hepatic IκB/NF-κB pathway (232). Furthermore, supplementation of Cr-histidinate in quails subjected to HS has been demonstrated to inhibit HSPs, specifically HSP60, HSP70, and HSP90 (233).

Beyond its antioxidant and anti-inflammatory roles, Cr is important for glucose metabolism and nutrient absorption, which are critical for maintaining bird performance under HS conditions. Chromium enhances insulin function by modulating chromodulin, GLUT-4, and uncoupling protein-3, leading to increased glucose and amino acid uptake and improved skeletal muscle mass (234). This metabolic regulation is crucial in birds subjected to HS, where energy balance and nutrient utilization are often compromised.

### 4.3 Dietary fat and fatty acids

The addition of fat to poultry diets increases the energy value of feed constituents and mitigates the effects of HS in birds. Heat stress accelerates gastrointestinal passage rates, but dietary fat addition slows the rate of food passage and enhances nutrient utilization (235). Additionally, higher-energy diets are effective in partially counteracting the harmful effects of HS, as fat metabolism generates lower heat increments compared to proteins and carbohydrates (236). The reduced thermogenic effect of fat, relative to carbohydrates and proteins, is attributable to differences in how these macronutrients are metabolized. Unlike carbohydrates, which undergo multiple metabolic transformations before being converted into body fat, fatty acids can be directly utilized in body fat synthesis without modification. This principle has led to the widespread practice of supplementing poultry diets with fat in hot climates, where the addition of fat helps increase dietary energy levels and reduce the detrimental impact of HS on bird performance.

Adding 5% fat to heat-stressed laying hens’ diets increased FI by 17% (158), while broilers fed a similar diet exhibited significant improvements in performance, nutrient digestibility, and carcass traits (237). Similarly, Attia et al. (238) stated that adding oil to high-protein diets helped alleviate the harmful effects of chronic HS on broiler performance, meat lipid composition, and physiological and immunological traits. Ghahremani et al. (239) showed that partially replacing dietary energy with soybean oil and increasing dietary energy concentration enhanced intestinal function and overall productivity in heat-stressed broilers. However, one notable outcome of fat supplementation is increased abdominal fat deposition in heat-stressed broilers (428).

Fatty acids, particularly short-chain and medium-chain fatty acids, are critical in stabilizing gut microbiota composition, regulating epithelial cell function, and alleviating oxidative stress caused by HS (Table 4). Short-chain fatty acids, with fewer than six carbon atoms, serve as key energy sources for gut microbiota and help maintain a healthy gut environment by lowering intestinal pH and inhibiting harmful bacterial growth (240). The improvement in gut epithelial structure helps protect against pathogenic bacterial invasion, thereby preventing intestinal permeability and enhancing overall growth performance in broilers.

**Table 4:** Effects of fatty acids and protein supplementation on heat-stressed poultry.

Species	Heat stress condition	Intervention and dosage	Key findings	References
Laying hens (Elma-Brown; 45 weeks)	36°C 63% RH 60 days	*Dunaliella salina* (omega-3 + carotenoids); 0.5, 1, and 1.5 g/kg of diet	Improved egg production and quality, antioxidant, and immune status	(280)
Broilers (Ross 308; 1-day-old)	35°C for 3 h on day 24	Fish oil; 2 mL/kg/bird/day starting from day 14	Reduced rectal temperature, interleukin-6, and corticosterone levels Increased superoxide dismutase and *heat shock protein 70* expression	(281)
Broilers (Cobb 500; 1-day-old)	33°C for 8 h from day 22–42 50% RH	Alpha-lipoic acid (500 mg/kg)	Upregulated intestinal antioxidants, tight-junction, and immune-related genes Enhanced the intestinal villus structure and enriched the beneficial microbiota	(282)
Broilers (Ross 308, Cobb 500; 1-day-old)	32°C; 55% RH for 6 h/day during 20–22 and 28–30 days of age	Standard-protein diet (22% CP) High-protein diet (24% CP and 12.97 MJ/kg) High-protein metabolizable energy diet (24% CP and 13.60 MJ/kg)	Compared to the standard-protein diet, the high-protein metabolizable energy diet improved BWG (4.6%), feed efficiency (6.3%), and physiological response for both strains Compared to the high-protein diet, the high-protein metabolizable energy diet increased protein and lipid digestibility High-protein metabolizable energy diet decreased the cloacal temperature (0.5%), respiration rate (2–3%), and heterophil:lymphocyte (1%)	(237)
Broilers (Hubbard; 1-day-old)	32°C from day 21–34 64% RH	Butyric acid (0.5 mg/kg)	Enhanced the abundance of beneficial bacteria Restored intestinal integrity Alleviated HS-induced mucosal damage	(121)
Broilers (Cobb; 1-day-old)	34°C for 12 h after 8 days of age	Olive oil (6.7%)	Reduced mitochondrial ROS synthesis and oxidative stress	(283)

BWG, body weight gain; CP, crude protein; h, hour; HS, heat stress; RH, relative humidity; ROS, reactive oxygen species.

### 4.4 Amino acids

Protein metabolism generates higher heat than fat and carbohydrates, thereby further increasing the adverse effect of HS in birds (241). While higher protein intake boosts heat production, reducing dietary protein can reduce BW. However, the adverse effects of supplying birds with low dietary protein can be countered through supplementation with crystalline limiting essential amino acids. Heat stress detrimentally impacts amino acids’ availability, transport, intestinal uptake, absorption, and utilization (145, 242). Therefore, the addition of balanced amino acids to low-protein diets can improve birds thermotolerance by lowering the energy cost for N excretion (243). In hot periods, diets lower in protein but enhanced with limiting amino acids yield better outcomes than high-protein diets.

Besides, maintaining amino acid balance and adequate amounts of limiting amino acids like lysine and arginine greatly helps in minimizing the effects of HS (166). Also, sulfur-containing amino acids, like methionine and cysteine, are essential for poultry nutrition. The supplementation of methionine reduced muscle oxidation and enhanced tissue antioxidant levels in broilers under HS (244). In another study, the supplementation of sulfur amino acids reduced chronic HS in broiler chickens by boosting antioxidant synthesis and protecting the permeability of the intestinal barrier (245, 246). According to Wu et al. (247), glutamine promotes growth performance, enhances gut development, and improves barrier function, including TJ protein expression. Additionally, Dai et al. (248) found that adding glutamine (0.5 and 1.0%) to the diets of broilers exposed to HS (28°C) mitigated losses in growth performance, carcass traits, meat quality, and meat color stability. Moreover, glutamine contributes significantly to stabilizing the intestinal microflora by enhancing the presence of *Lactobacillus* and *Bifidobacterium* in the cecum while decreasing the levels of *Clostridium perfringens* and *E. coli* in broiler chickens exposed to HS.

Glycine, a conditionally essential amino acid in poultry, is also crucial for improving production performance and reducing oxidative stress and intestinal dysfunction in heat-stressed birds (242, 249). Some non-essential amino acids and their derivatives, like betaine, taurine, L-citrulline, and L-theanine, can help reduce HS in poultry (242). These compounds exhibit strong biological effects, acting as anti-stress agents, antioxidants, anti-inflammatory agents, immune system boosters, and gut stimulants when administered to poultry experiencing HS.

### 4.5 Electrolytes

Under high temperatures, birds pant, causing a disruption in acid–base homeostasis and resulting in respiratory alkalosis. Electrolyte salts such as sodium chloride, sodium bicarbonate, ammonium chloride, potassium chloride, and potassium sulfate can be used for the restoration of the acid–base concentration in the blood (250, 251) as they dissociate to release electrolytes (ions). For every 1°C rise in ambient temperature, there is a corresponding 1.4% decrease in average daily FI and a 2.1% decline in average daily weight gain (252), while the demand for water intake is increased. At 38°C, chickens consume four times more water than at 21°C (253). Drinking more water aids in cooling the body and enhances heat dissipation, helping to alleviate the symptoms of heat exhaustion, especially when electrolytes like Na^+^, K^+^, and Cl^−^ salts are supplemented in the water (254). Electrolyte supplementation in poultry drinking water restores essential nutrients that balance blood pH levels (32).

Electrolytes are vital for poultry health, especially during HS, and it is advisable to maintain adequate DEB levels under such conditions (255). The effect of electrolyte supplementation is contingent upon the DEB. Research indicates that moderate DEB values, ranging from 120 to 240 mEq, is beneficial to the physiological responses of heat-stressed broiler chickens (140). Heat stress causes hyperventilation, which disrupts the blood’s acid–base equilibrium, leading to respiratory alkalosis that hinders broiler chickens’ growth and laying hens’ egg production and eggshell quality. Farfán et al. (256) found that broilers under HS maintained a balanced electrolyte level of 240 mEq when provided with mineral-enriched water. This supplementation of minerals is associated with a decrease in metabolic heat hyperventilation. Bryden et al. (257) revealed that laying hens under HS showed improvements in both egg production and quality when given 0.5% hydrochloric acid in their drinking water.

Sodium bicarbonate is commonly utilized at high temperatures (258), and adding this salt to the diets of heat-stressed broiler chickens enhanced their performance (259). Also, providing NaHCO_3_ to laying hens can enhance eggshell quality, provided the hens have access to feed during the eggshell formation phase, facilitated by continuous light exposure (136). Water intake during HS is crucial, and incorporating electrolytes into poultry diet or drinking water helps to enhance water consumption (144), which improves performance. Supplementing 1% NH_4_Cl or 0.5% NaHCO_3_ (260) and 1.5 to 2.0% K in the form of KCl (261) can partially alleviate growth suppression in broilers. Feeding chickens 1.5–2.0% K from KCl reduced FCR during chronic HS. Additionally, supplementing drinking water with 0.2% NH_4_Cl, 0.150% KCl, and carbonated water further boosted chicken performance (144).

### 4.6 *In ovo* feeding

Recently, manipulation during embryogenesis, including thermal conditioning and *in ovo* feeding has been explored to combat HS. *In ovo* feeding (IOF) entails injecting nutrients or bioactive compounds into fertilized eggs during mid or late embryogenesis (around day 12–18) to support the embryo and early chick development and improve physiological performance (262). The delivery route for the nutrients varies and is dependent on time (262). During the early incubation phase, the air sac is chosen as the injection site (263). In contrast, at later stages, the amnion becomes the preferred route for injection because developing chicks can ingest amniotic fluid by that time (264).

*In ovo* injection of sulfur amino acids, cysteine (3.4 mg) and methionine (5.9 mg), during a high-temperature incubation (39.6°C for 6 h daily) from embryonic day (ED) 10 to 18 significantly downregulated stress markers (*HSP90* and corticosterone) and elevated antioxidant defenses (total antioxidant capacity, glutathione, and glutathione:oxidized glutathione) in tissues (265). In the same study, IOF increased the intestinal villus area in the broiler embryos (265). Similarly, IOF of vitamin C (3 mg/egg) into the yolk at ED 11 reduced embryonic mortality and improved hatchability under an acute HS 40°C challenge (266). Mechanistically, IOF primes the chick’s antioxidant and immune systems and stimulates gut development before hatch.

## 5 Future direction

While significant progress has been made in understanding the effects of HS on poultry gut health and performance, and the potential of nutritional supplements to alleviate these effects, several knowledge gaps remain. The optimal dosages and combinations of vitamins, minerals, amino acids, and electrolytes are yet to be proven. Further research is needed to determine the precise quantities required to achieve maximum thermotolerance and to explore the interactions between different supplements and their synergistic effects. Additionally, most studies focused on short-term outcomes, so the long-term effects of continuous supplementation on poultry health and productivity are poorly understood. Future research should address the sustainability of these interventions, particularly in large-scale commercial production systems, to assess their cost-effectiveness and long-term feasibility.

Another area for exploration is the role of gut microbiota in HS responses. While some studies have shown that HS negatively impacts gut integrity, more research is needed to understand the mechanism behind specific nutritional supplements influencing the gut microbiota to mitigate these effects. Addressing these gaps will improve poultry resilience to HS and enhance global poultry production and food security in the face of climate change.

## 6 Conclusion

Heat stress remains a critical challenge in poultry production, with significant implications for bird health, welfare, and productivity. As climate change intensifies, the poultry industry faces growing pressure to mitigate the adverse impacts of rising temperatures on poultry. The physiological responses to HS, including altered metabolism, reduced FI, impaired gut integrity, and oxidative stress, necessitate effective mitigation strategies. Nutritional supplementation has proven to be an efficient approach to enhancing poultry resilience against HS. Research demonstrates that vitamins, minerals, fat, amino acids, and electrolytes can mitigate some of the adverse effects of HS by improving growth, enhancing antioxidant defense, modulating immune responses, and maintaining gut health. Vitamins A and C, along with minerals such as selenium, zinc, and chromium, are particularly effective in reducing oxidative stress, supporting immune responses, and stabilizing metabolic functions. These supplements have proven to improve feed intake, weight gain, and egg production, while also mitigating the adverse effects on gut integrity under HS conditions. Fat and amino acid supplementation, especially in combination with other dietary interventions, has been identified as beneficial for improving nutrient digestibility and enhancing poultry thermotolerance. Furthermore, electrolyte supplementation supplies minerals and plays a critical role in restoring acid–base balance and supporting hydration during heat exposure.

While current studies provide valuable insights, further research is needed to optimize supplement combinations, determine optimal dosages, and assess long-term effects in commercial poultry production. Integrating targeted nutritional strategies with improved management practices can significantly enhance poultry performance, gut health, and adaptability to rising global temperatures, ensuring sustainable production and food security.
